# Impact of transvenous cardiac implantable electronic devices in chronic hemodialysis patients: a single-center, observational comparative study

**DOI:** 10.1186/s12882-018-1095-y

**Published:** 2018-10-20

**Authors:** Seonjeong Jeong, Gi Byoung Nam, Jai Won Chang, Min-Ju Kim, Youngjin Han, Tae-Won Kwon, Yong-Pil Cho

**Affiliations:** 1 Division of Vascular Surgery, Department of Surgery, University of Ulsan College of Medicine, Asan Medical Center, 88, Olympic-ro 43-gil, Seoul, 05505 Republic of Korea; 2 Division of Cardiology, Department of Internal Medicine, University of Ulsan College of Medicine, Asan Medical Center, Seoul, Republic of Korea; 3 Division of Nephrology, Department of Internal Medicine, University of Ulsan College of Medicine, Asan Medical Center, Seoul, Republic of Korea; 4 Department of Clinical Epidemiology and Biostatistics, University of Ulsan College of Medicine, Asan Medical Center, Seoul, Republic of Korea

**Keywords:** Artificial pacemaker, Implantable defibrillators, Renal dialysis

## Abstract

**Background:**

We investigated the impact of a transvenous cardiac implantable electronic device (CIED) placement on outcomes and arteriovenous vascular access (VA) patency among chronic hemodialysis patients.

**Methods:**

This is a single-center, observational comparative study between chronic hemodialysis patients with ipsilateral and contralateral CIED and VA. Forty-two consecutive patients who underwent both CIED placement and upper-extremity VA for hemodialysis, regardless of the sequence and time interval between these 2 procedures, were identified between January 2001 and December 2017. Patients with ipsilateral (*n* = 22, 52%, the ipsilateral group) and contralateral (*n* = 20, 48%, the contralateral group) CIED and VA were compared retrospectively; the primary outcome was any-cause mortality and cardiac mortality or the composite of any systemic complications, defined as central venous stenosis or occlusion, any device infections or tricuspid regurgitation; the secondary outcome was CIED or VA malfunction.

**Results:**

During the median follow-up period of 101 months, primary outcome incidence was significantly higher in the ipsilateral group than the contralateral group (73% vs 40%, *P* = 0.03), although the incidences of any-cause mortality (*P* = 0.28) and cardiac mortality (*P* > 0.99) were similar between the groups. Secondary outcome incidence did not differ significantly between the 2 groups (55% vs 30%, *P* = 0.36). Kaplan–Meier survival analysis revealed similar primary and secondary VA patency rates in both groups. On subgroup analysis, patients with upper arm VA had similar primary and secondary patency to those with forearm VA.

**Conclusions:**

Despite some notable limitations of the study, the retrospective study design and small sample size, we found that the any-cause mortality incidence and VA patency did not differ between the 2 groups, but primary outcome incidence was significantly higher among patients with ipsilateral CIED and VA.

## Background

In patients with chronic kidney disease (CKD), regardless of the predialysis or dialysis stage, cardiovascular disease is a major cause of mortality; more than one-third of cardiac deaths are attributable to arrhythmias [1–4]. Although the precise prevalence of cardiac implantable electronic devices (CIEDs) in chronic hemodialysis patients is unknown, there is a frequent need for CIED placement, such as with a permanent pacemaker (PM) or implantable cardioverter defibrillator (ICD) for arrhythmia treatment or sudden cardiac death prevention [1–3]. Despite a substantial complication rate associated with transvenous leads and despite recently developed alternative options—such as subcutaneous ICD, epicardial leads, and leadless PM—the most common method of CIED insertion is still transvenous placement of electrical leads, with implantation of an impulse generator in a subcutaneous pocket [2, 4]. For patients with CKD requiring hemodialysis and a CIED, the general recommendation is for arteriovenous vascular access (VA) on the upper limb contralateral to the CIED [2, 5]. However, it is not always feasible to adhere to this recommendation due to the limited availability of VA sites and the frequent (and sometimes inevitable) necessity for VA revision due to suboptimal patency rates [5].

This study investigated the impact of transvenous CIEDs on long-term clinical outcomes in chronic hemodialysis patients and compared outcomes among patients with ipsilateral VA vs contralateral VA, relative to CIED location. We also investigated the effects of CIEDs on VA patency in these patients.

## Methods

### Study design and population

In this single-center, retrospective, observational cohort study, we analyzed data extracted from patient medical records. Our hospital’s institutional review board (2018–0442) approved the study protocol and waived the need for informed consent. Between January 2001 and December 2017, 42 consecutive chronic hemodialysis patients who underwent both CIED and upper-extremity VA placement, regardless of the sequence and time interval between these 2 procedures, were included. They comprised 1.9% of all patients undergoing CIED placement (1564 PMs and 600 ICDs) at our hospital during the same period.

All patients underwent preoperative assessment with physical examination, duplex ultrasound, or both, and all VA creation procedures were performed using local anesthesia and as described elsewhere [6, 7]. VAs were categorized as arteriovenous fistula (AVF) vs prosthetic arteriovenous graft (AVG) according to VA type, or forearm vs upper arm according to VA location. There were no cases of maturation failure, and all included VAs provided adequate dialysis for at least 3 dialysis sessions without further intervention. All included patients were stratified into 2 groups according to the side of CIED placement relative to upper-extremity VA location as follows: the ipsilateral group (ipsilateral CIED and VA) and the contralateral group (contralateral CIED and VA).

### Study outcomes and follow-up

The ipsilateral and contralateral groups were retrospectively analyzed and compared with regard to long-term clinical outcomes. The primary outcome was any-cause mortality and cardiac mortality or the composite of any systemic complication, defined as ipsilateral or contralateral central venous (CV) stenosis or occlusion, any device infections, and newly developed or aggravated tricuspid regurgitation. The secondary outcome was CIED or VA malfunctions. The central veins were defined as the subclavian vein, brachiocephalic vein, or superior vena cava [8]. In our institution, angiographic evaluations were performed if any of the following signs and symptoms suspicious of CV stenosis or occlusion appeared: decreased or absent thrill, difficult cannulation, prolonged bleeding time after dialysis, development of collateral veins, persistently elevated dynamic venous pressures unexplained by needle position or size, high recirculation rate (> 10%), arm swelling, pain, or neurologic symptoms [5, 9, 10]. The diagnosis of CV stenosis or occlusion was confirmed by fistulogram in this study. Primary patency was defined as the interval from the time of VA placement until any intervention designed for maintenance or reestablishment of VA function, VA failure, or last follow-up, whichever occurred first. Secondary patency was defined as the interval from the time of VA placement until the abandonment of the VA for any reason, regardless of the number of subsequent interventions. Follow-up data were retrieved from individual medical records or follow-up physicians, and clinical outcomes were documented to the date of last follow-up. In this analysis, only the first event of each outcome was included. This study recorded individual demographics, risk factors of interest, and other data. The study outcomes were retrospectively analyzed in an Excel (Microsoft Corp., Redmond, WA, USA) database.

### Statistical analysis

Categorical variables are reported as frequencies and percentages and continuous variables as medians and ranges. Comparisons of categorical variables were made using the chi-squared test or Fisher’s exact test, where appropriate. Comparisons of continuous variables were made using Student’s t*-*test or the Mann–Whitney U test, where appropriate. Survival curves of primary outcome-free survival rates and VA patency rates were constructed using Kaplan–Meier estimates and were compared using the log-rank test. *P* < 0.05 was considered statistically significant. Statistical analyses were performed with SPSS Version 21.0 (SPSS Inc., Chicago, IL, USA).

## Results

Among the 42 records included in the analysis, there were 22 patients (52%) in the ipsilateral group and 20 patients (48%) in the contralateral group; this included 35 PMs (83%) and 7 ICDs (17%). The most common indications for CIED implantation were sick sinus syndrome (*n* = 19, 45%) and atrioventricular (AV) block (*n* = 10, 24%). The demographic data, risk factors, and clinical characteristics, stratified into the ipsilateral or contralateral CIED and VA, are summarized in Table 1. There were no significant differences in patient characteristics between the ipsilateral and contralateral groups, except in the proportion of patients undergoing CIED implantation after VA placement and the presence of a right-sided CIED. Previous use of a CV catheter for hemodialysis was noted in 33 patients (79%), and the median duration of hemodialysis via CV catheter was 3 months (range, 1–38 months). The proportions and durations of previous CV catheter use were similar between the 2 groups. Upper arm VA was noted in 9 patients (21%), and there was no significant difference in the type of VA (AVF vs AVG) between the 2 groups. Sixteen patients (38%) had CIEDs implanted after VA placement. The majority of CIEDs (*n* = 32, 76%) were on the left side, and 10 CIEDs were on the right side. Most of the right-sided CIEDs (9 of 10) were implanted after VA placement on the left upper limb. The proportions of patients with CIED implantation after VA placement (23% vs 55%, *P* = 0.03) and right-sided CIEDs (5% vs 45%, *P* = 0.03) were significantly higher in the contralateral group.

**Table 1:** Demographic and clinical characteristics of the patients with ipsilateral vs contralateral CIED and VA

	Total	Ipsilateral	Contralateral	*P* value
	(*n* = 42)	(*n* = 22)	(*n* = 20)	
Age (years)	68 (36–83)	65 (45–83)	70 (36–83)	0.50
Male sex	23 (55)	11 (50)	12 (60)	0.52
Risk factor				
Diabetes mellitus	26 (62)	14 (67)	12 (60)	0.81
Hypertension	34 (81)	17 (77)	17 (85)	0.70
Coronary artery disease	18 (43)	7 (32)	11 (55)	0.37
CIED				
Pacemaker	35 (83)	18 (82)	17 (85)	> 0.99
ICD	7 (17)	4 (18)	3 (15)	
Transvenous route, CIED lead				
Cephalic vein	36 (86)	18 (82)	18 (90)	0.67
Subclavian vein	6 (14)	4 (18)	2 (10)	
Cause of ESRD				
Diabetes mellitus/Hypertension	31 (74)	14 (64)	17 (85)	0.17
Acute kidney injury	4 (10)	2 (9)	2 (10)	NA
Chronic glomerulonephritis	3 (7)	3 (14)	0	NA
Others	4 (10)	3 (14)	1 (5)	NA
CV catheter^a^	33 (79)	16 (73)	17 (85)	0.46
Duration (months)^b^	3 (1–38)	3 (1–38)	2.5 (1–10)	0.42
Type of VA				
AVF	28 (67)	14 (64)	14 (70)	0.66
Wrist side-to-end	15	6	9	
Forearm side-to-end	13	8	5	
AVG	14 (33)	8 (36)	6 (30)	
Forearm U-loop	5	3	2	
Upper arm straight	9	5	4	
Sequence of CIED and VA				
CIED after VA	16 (38)	5 (23)	11 (55)	0.03
Right sided CIED	10 (24)	1 (5)	9 (45)	0.03
Time interval (months)^c^	25 (1–117)	7 (1–40)	29 (4–117)	0.14

Continuous data are expressed as medians (ranges); categorical data are expressed as a number (%)

*AVF* arteriovenous fistula, *AVG* prosthetic arteriovenous grafting, *CIED* cardiac implantable electronic device, *CV* central venous, *ESRD* end-stage renal disease, *ICD* implantable cardioverter defibrillator, *NA* not applicable, *VA* vascular access

^a^ CV catheter for hemodialysis

^b^ Duration of hemodialysis via CV catheter

^c^ Time interval between CIED placement and VA creation

**Fig. 1:**
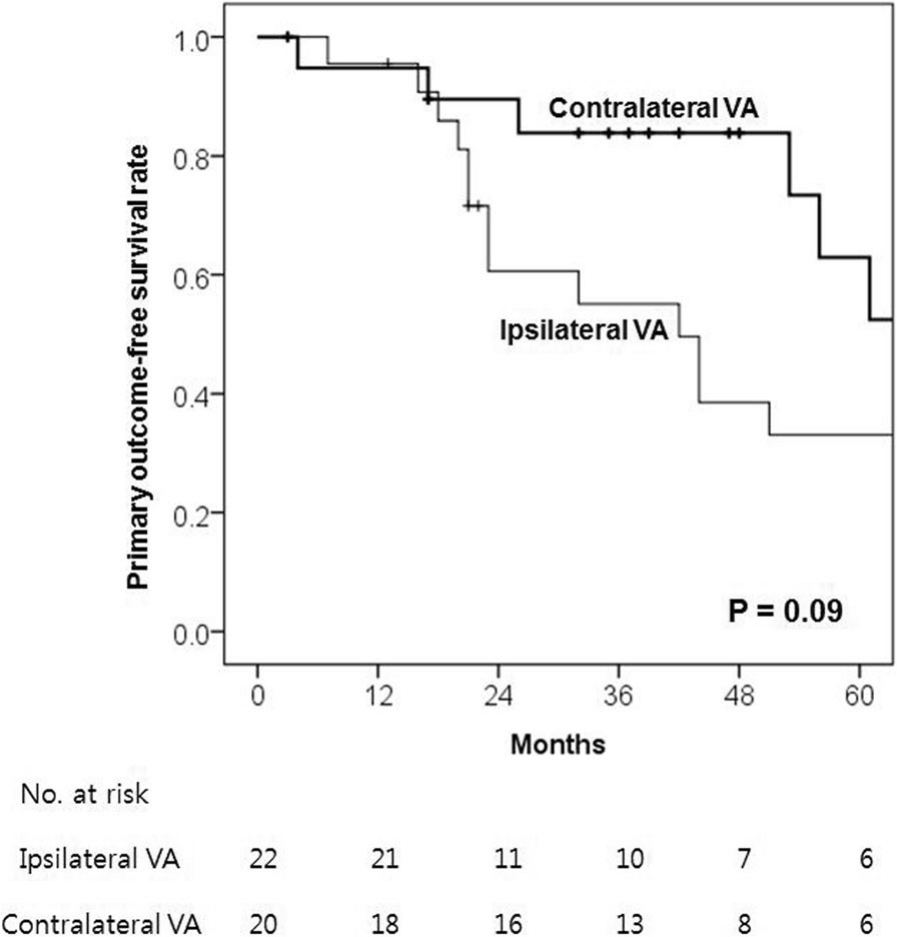
Kaplan–Meier estimates of primary outcome-free survival rate between the patients with ipsilateral vs contralateral vascular access (VA) and cardiac implantable electronic devices

**Fig. 2:**
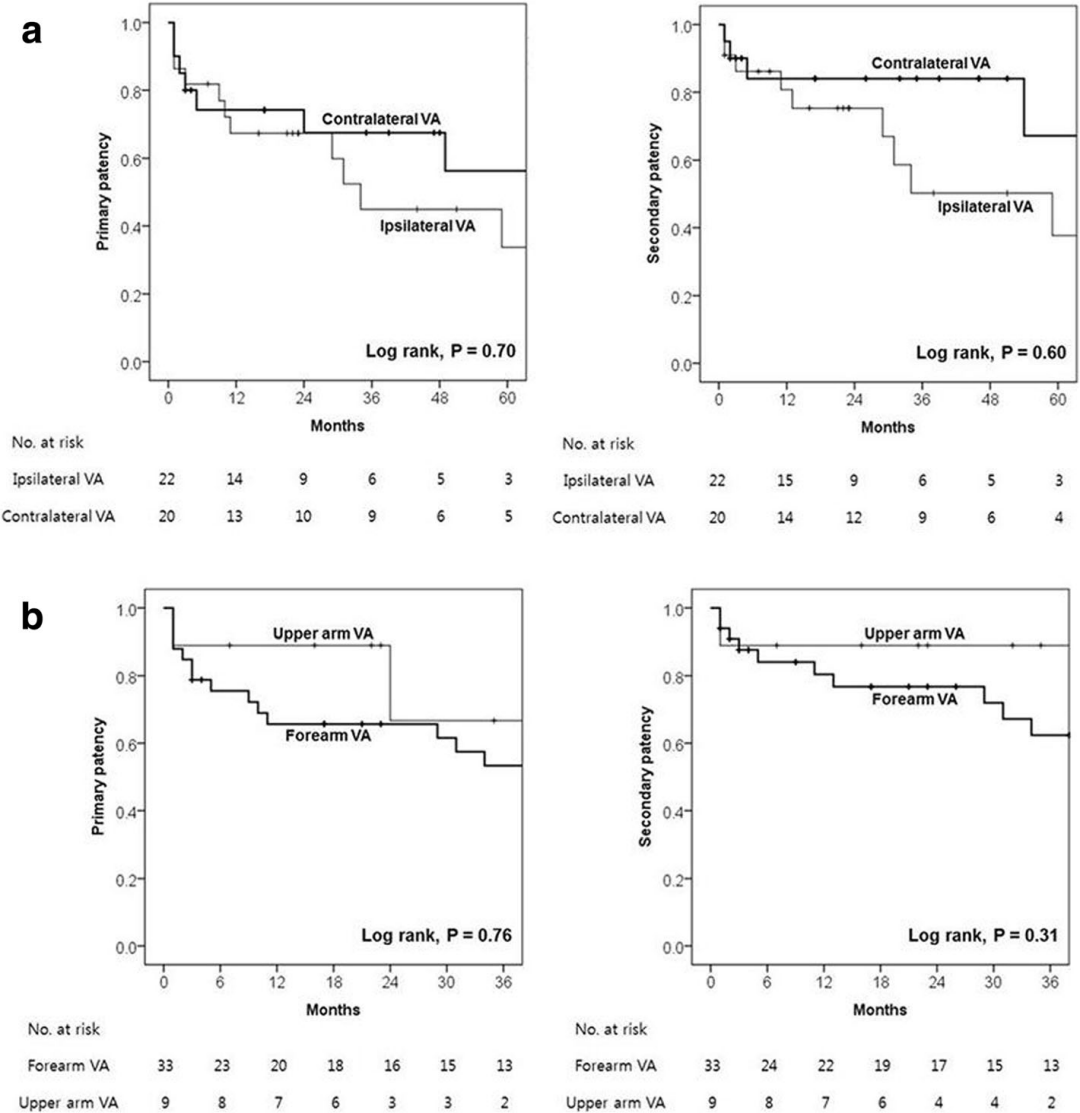
Kaplan–Meier estimates of primary and secondary patency rates (**a**) between the patients with ipsilateral vs contralateral vascular access (VA) and cardiac implantable electronic devices and (**b**) between forearm vs upper arm VA

The median follow-up period was 101 months (mean, 100 months; range, 5–263 months). There were three patients with short-term follow-up less than 12 months: two mortality cases, at 4 and 7 months, respectively, and one lost to follow-up at 3 months. Long-term clinical outcomes of the study sample are presented in Table 2. Primary outcome incidence was significantly higher in the ipsilateral group than the contralateral group (73% vs 40%, *P* = 0.03). Although the incidences of any-cause mortality (41% vs 25%, *P* = 0.28) and cardiac mortality (18% vs 15%, *P* > 0.99) were similar between the 2 groups, there was a higher incidence of systemic complications in the ipsilateral group, and this difference was clinically significant (55% vs 25%, *P* = 0.051). CV stenosis ipsilateral to the CIED was noted in 7 of the 42 patients (17%), and there was a higher (although nonsignificant) incidence of CV stenosis in the ipsilateral group (27% vs 5%, *P* = 0.08). There were no device-related infections in either group during the study period. On Kaplan–Meier survival analysis, the primary outcome-free survival rate showed a slightly increasing trend in the contralateral group compared with the ipsilateral group (*P* = 0.09) (Fig. 1). Secondary outcome incidence did not differ significantly between the 2 groups (55% vs 30%, *P* = 0.36). Kaplan–Meier survival analysis revealed similar primary (*P* = 0.70) and secondary (*P* = 0.60) VA patency rates in the 2 groups (Fig. 2). Primary and secondary VA patency rates are summarized in Table 3. The median primary and secondary patency durations for ipsilateral and contralateral VA and CIED were 34 and 65 months, and 59 months and 78 months, respectively; although the proportion of patients undergoing CIED implantation after VA placement was significantly higher in the contralateral group, these differences were not statistically significant. Our subgroup analysis was based on VA location, and it showed that primary (*P* = 0.76) and secondary (*P* = 0.31) patency rates were similar between patients with forearm vs upper arm VA.

**Table 2:** Clinical outcomes of the patients with ipsilateral vs contralateral CIED and VA

	Total	Ipsilateral	Contralateral	*P* value
	(*n* = 42)	(*n* = 22)	(*n* = 20)	
Follow-up duration (months)	101 (5–263)	101 (20–263)	83 (5–146)	0.70
Mean (months)	100	111	86	
Primary outcome	24 (57)	16 (73)	8 (40)	0.03
Any-cause mortality	14 (33)	9 (41)	5 (25)	0.28
Cardiac mortality	7 (17)	4 (18)	3 (15)	> 0.99
Heart failure	3	1	2	
Myocardial infarction	1	1	0	
Recurrent VT	2	1	1	
Cardiopulmonary failure	1	1	0	
Septic shock^a^	2	1	1	
Others	5	4	1	
Systemic complications	17 (41)	12 (55)	5 (25)	0.051
Ipsilateral CV stenosis^b^	7 (17)	6 (27)	1 (5)	0.08
Contralateral CV stenosis^c^	2 (5)	–	2 (10)	NA
Tricuspid regurgitation^d^	10 (24)	7 (32)	3 (15)	0.28
Secondary outcome	22 (52)	12 (55)	10 (30)	0.36
VA malfunction	22 (52)	12 (55)	10 (30)	0.36
Occlusion/stenosis	16	7	9	
Infection	1	1	0	
Others	5	4	1	
CIED malfunction	0	0	0	NA
Overall survival (months)	117 (4–143)	93 (7–143)	136 (4–137)	0.25
Mean (months)	94	82	107	

Continuous data are expressed as medians (ranges); categorical data are expressed as a number (%)

*CIED* cardiac implantable electronic device, *CV* central venous, *NA* not applicable, *VA* vascular access, *VT* ventricular tachycardia

^a^ Septic shock not related to CIED or VA

^b^ CV stenosis ipsilateral to the CIED lead

^c^ CV stenosis contralateral to the CIED lead

^d^ Newly developed or aggravated tricuspid regurgitation

**Table 3:** Primary and secondary patency of the VA stratified by the sides and locations of VA

		Ipsilateral vs contralateral VA			Forearm vs upper arm VA		
	Total	Ipsilateral VA	Contralateral VA	*P* value	Forearm	Upper arm	*P* value
	(*n* = 42)	(*n* = 22)	(*n* = 20)		(*n* = 33)	(*n* = 9)	
CIED after VA	16 (38.1)	5 (22.7)	11 (55)	0.03	13 (39.4)	3 (33.3)	0.74
Time interval (months)	25 (1–117)	7 (1–40)	29 (4–117)	0.14	22 (1–117)	29 (8–61)	0.32
Primary patency (months)	59 (1–136)	34 (1–117)	65 (1–136)	0.70	59 (1–136)	49 (1–49)	0.76
1 year (%)	71	67	74	0.72	66	89	0.29
3 years (%)	57	45	68	0.39	53	67	0.39
5 years (%)	46	34	56	0.36	NA	NA	NA
Secondary patency (months)	65 (1–136)	59 (1–117)	78 (1–136)	0.60	59 (1–136)	45 (1–51)	0.31
1 year (%)	82	81	84	0.81	80	89	0.61
3 years (%)	67	50	84	0.12	62	89	0.31
5 years (%)	52	38	67	0.15	NA	NA	NA

Continuous data are expressed as medians (ranges)

*CIED* cardiac implantable electronic device, *NA* not applicable, *VA* vascular access

## Discussion

The prevalence of CKD is estimated to be 8–16% worldwide, and regardless of the predialysis or dialysis stage, cardiovascular disease is the leading cause of mortality in a majority of these patients [2, 11]. According to the United States Renal Data System database, more than one-third of cardiac deaths are attributable to arrhythmias [1, 2, 4], and therefore the American College of Cardiology /American Heart Association guidelines recommend CIEDs in selected patients for the prevention of sudden cardiac death [1–4]. Hence, the number of patients with CKD requiring CIEDs and VA has been increasing over the years [1, 4, 8, 12]. Transvenous leads from a CIED are known to cause or contribute to adverse events that are of particular concern for the hemodialysis population. They can cause CV stenosis, contribute to CIED-related infective endocarditis, and they carry the risk of rare tricuspid regurgitation by valve adhesion, perforation, or entanglement [4, 5, 8, 13, 14]. In patients with CKD requiring CIED and VA placement, it is generally recommended to place arteriovenous VA on the contralateral upper limb relative to a CIED, in addition to the avoidance of CV catheter use for hemodialysis [2, 5]. However, controversy exists regarding the best management strategy in these patients because there is relatively little known about variations in outcomes according to VA location relative to CIED leads. Although the National Kidney Foundation KDOQI Guidelines, in 2000, recommended against VA on the ipsilateral side of an existing CIED lead, this is not feasible in some patients even if other options have been exhausted. The 2006 KDOQI update offered no such recommendation, in the absence of supporting clinical data [2, 5, 8, 12].

The results of our current study indicate that the incidence of any systemic complications, especially CV stenosis ipsilateral to the CIED lead, was higher in patients with ipsilateral CIED and VA placement (with clinical significance), whereas any-cause mortality and cardiac mortality did not differ between the 2 groups. In contrast to a recent study that reported poor outcomes for VA with ipsilateral CIED leads [12], our analysis showed similar median primary and secondary patency for VA ipsilateral vs contralateral to CIED leads, and there was no significant difference in patency rate between patients with forearm vs upper arm VA.

Considering that the long-term patency of a functioning VA is extremely important for quality of life and longevity in patients with CKD requiring dialysis through an upper limb arteriovenous VA, CV stenosis is considered a serious complication contributing to dialysis VA dysfunction [4]. CV stenosis, reported to occur in as many as 60% of non-hemodialysis-dependent patients with a CIED, is generally asymptomatic [15]. However, chronic hemodialysis patients with ipsilateral CIED and VA placement are at particular risk of developing symptoms, such as edema of the face, neck, breast, shoulder, and arm, on account of progressive CV stenosis. Symptomatic stenosis or occlusion has been reported to be more frequently associated with high-flow VAs, because of the abnormal hemodynamics that accompanies increased blood flow through the central veins [9, 16]. Additionally, it has been reported that elevated venous pressures and high recirculation rates represent arteriovenous VA dysfunction, leading to reduce patency in patients with transvenous CIEDs [12, 16]. CV stenosis can implicate the subclavian, brachiocephalic veins, and the superior vena cava [4]. Despite these previous reports, the association between a transvenous CIED and clinical outcomes among chronic hemodialysis patients remains controversial. In our study, the incidence of CV stenosis or occlusion was markedly low compared to previous studies [9]; this may be explained by the fact that our study included only the symptomatic cases confirmed by fistulogram.

The risk of device-related infections, including endocarditis, in chronic hemodialysis patients with transvenous CIEDs, has been reported to be higher than that among members of the general population with CIEDs [17]. Not only do CIED infections—especially those associated with ICDs—reduce some of the immediate mortality benefits brought about by arrhythmia prevention, but they also lead to increased healthcare costs, prolonged hospital stays, and higher medium-term mortality rates among chronic hemodialysis patients [18]. Opelami et al. [19] analyzed CIED infection-related hospitalizations among chronic hemodialysis patients and found that dialysis patients had a significantly higher in-hospital mortality and longer median length of hospital stay compared to the non-dialysis group. This may have been due to the deteriorated immune function in individuals on chronic hemodialysis and incompletely endothelialized transvenous leads, which limit a localized reaction and increase the risk of systemic infection for these patients [2]. An episode of bacteremia, most commonly caused by *Staphylococcus* species, can trigger the hematogenous spread of the infection to the CIED and can also cause transvenous lead-associated endocarditis, eventually leading to removal of the CIED and the arteriovenous VA [13]. According to a meta-analysis by Polyzos et al. [20], the most substantial patient-related risk factors associated with CIED infection are CKD, diabetes, corticosteroid use, chronic obstructive pulmonary disease, history of previous device infection, and skin disorders. Procedure-related factors include postoperative hematomas, reintervention for lead dislodgement, device replacement or revision, lack of antibiotic prophylaxis, temporary pacing, inexperienced operators, and procedure duration [2]. In chronic hemodialysis patients with transvenous CIEDs, liberal use of proper periprocedural antibiotic therapy is warranted to reduce the risk of device-related infections, if indicated based on prior microbiologic data. In our analysis, there were no device-related infections during the study period, and 2 deaths due to sepsis were not associated with CIED- or arteriovenous VA-related infections.

This study had some limitations. First, because this research was retrospective, it may have been affected by selection and information biases. Hence, the incidence of CV stenosis and other complications may have been underestimated. We could not analyze asymptomatic CV stenosis. Furthermore, the decisions to perform CIED implantation before or after VA placement and side selection were mainly made by the physician, based on the expected level of technical procedural difficulty. Second, our study cohort consisted only of subjects of Asian descent; thus, our findings should be cautiously interpreted when considering other ethnic groups. Third, the small sample size in this single-center cohort limits the overall generalizability of our results. Additional large cohort studies are required to establish the association between transvenous CIEDs and the clinical outcomes of chronic hemodialysis patients.

## Conclusions

The most common method of CIED placement is still with transvenous electrical leads and implantation of an impulse generator in a subcutaneous pocket, and the presence of electrical leads in the central veins carries a serious risk of vascular and infectious complications; this demands an individualized approach to VA. However, in patients with CKD requiring a CIED and VA, the management strategy has not yet been standardized, owing to a lack of supporting clinical data. Although our limited experience involved only a small number of patients, and there were no significant differences in the median primary and secondary patency rates between VA ipsilateral and contralateral to CIED leads, our analysis suggested that the incidence of systemic complications or mortality was significantly higher in chronic hemodialysis patients with CIEDs ipsilateral to their VA location. Whenever feasible, it is preferable to avoid ipsilateral CIED and VA. Our results could provide valuable background evidence for further large cohort studies to establish the best management strategy for these patients.

## Abbreviations

AV: Atrioventricular
AVF: Arteriovenous fistula
AVG: Prosthetic arteriovenous graft
CIED: Cardiac implantable electronic device
CKD: Chronic kidney disease
CV: Central venous
ICD: Implantable cardioverter defibrillator
PM: Permanent pacemaker
VA: Vascular access
